# Supplementary figures and images for: Noradrenaline and acetylcholine shape functional connectivity organization of NREM substages: An empirical and simulation study
Source: PLoS Comput Biol. 2025 Oct 28;21(10):e1012852. doi: 10.1371/journal.pcbi.1012852 (PMC12585100; doi:10.1371/journal.pcbi.1012852)

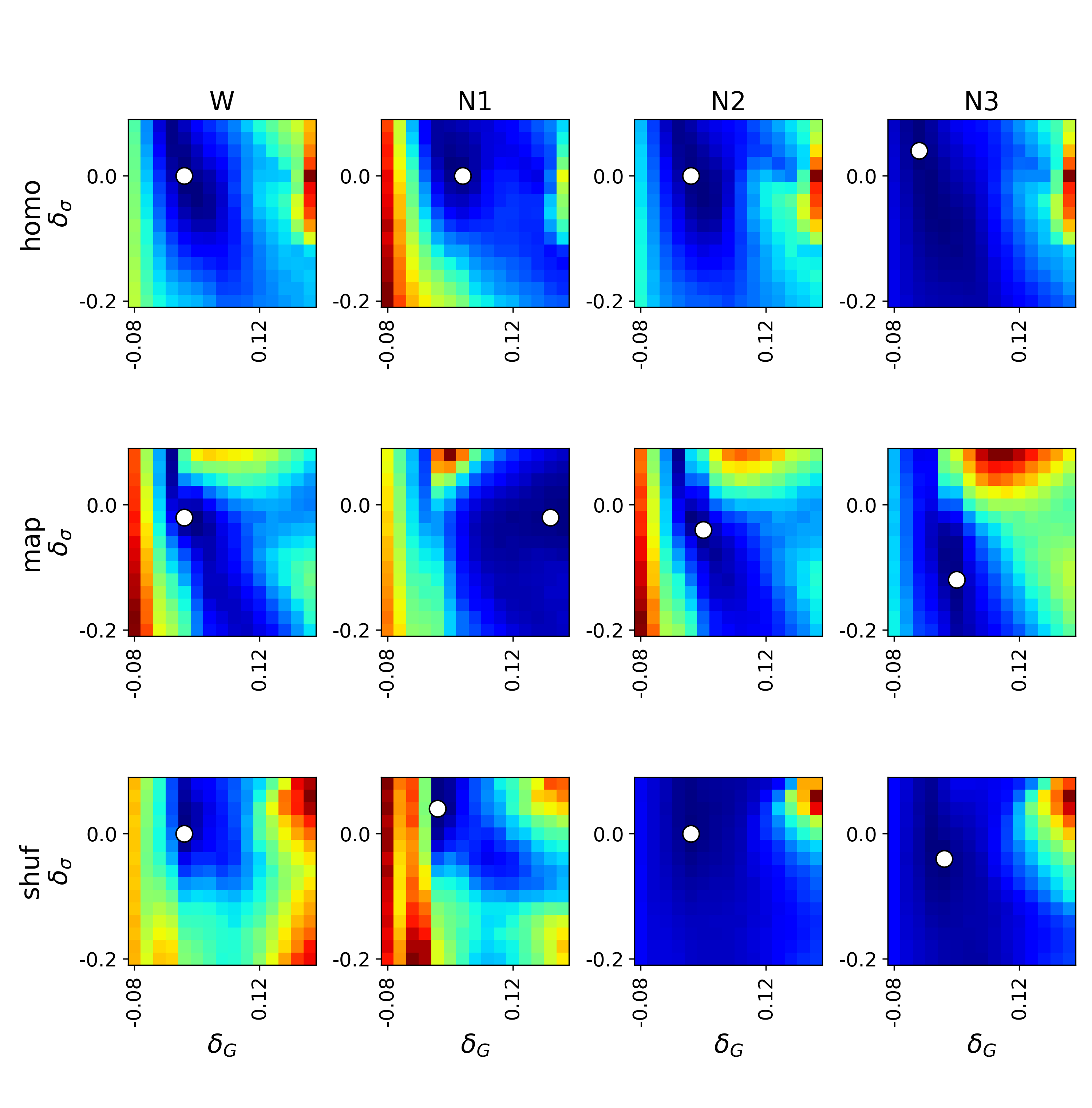

Supplement: S1 Fig — Variations are taken from the homogeneous optimal of W (G= 0.14, σ = 7.7). Here, lower values (blue) indicate a better fit the empirical FC matrix of each state. (TIFF) [file pcbi.1012852.s001.tiff]

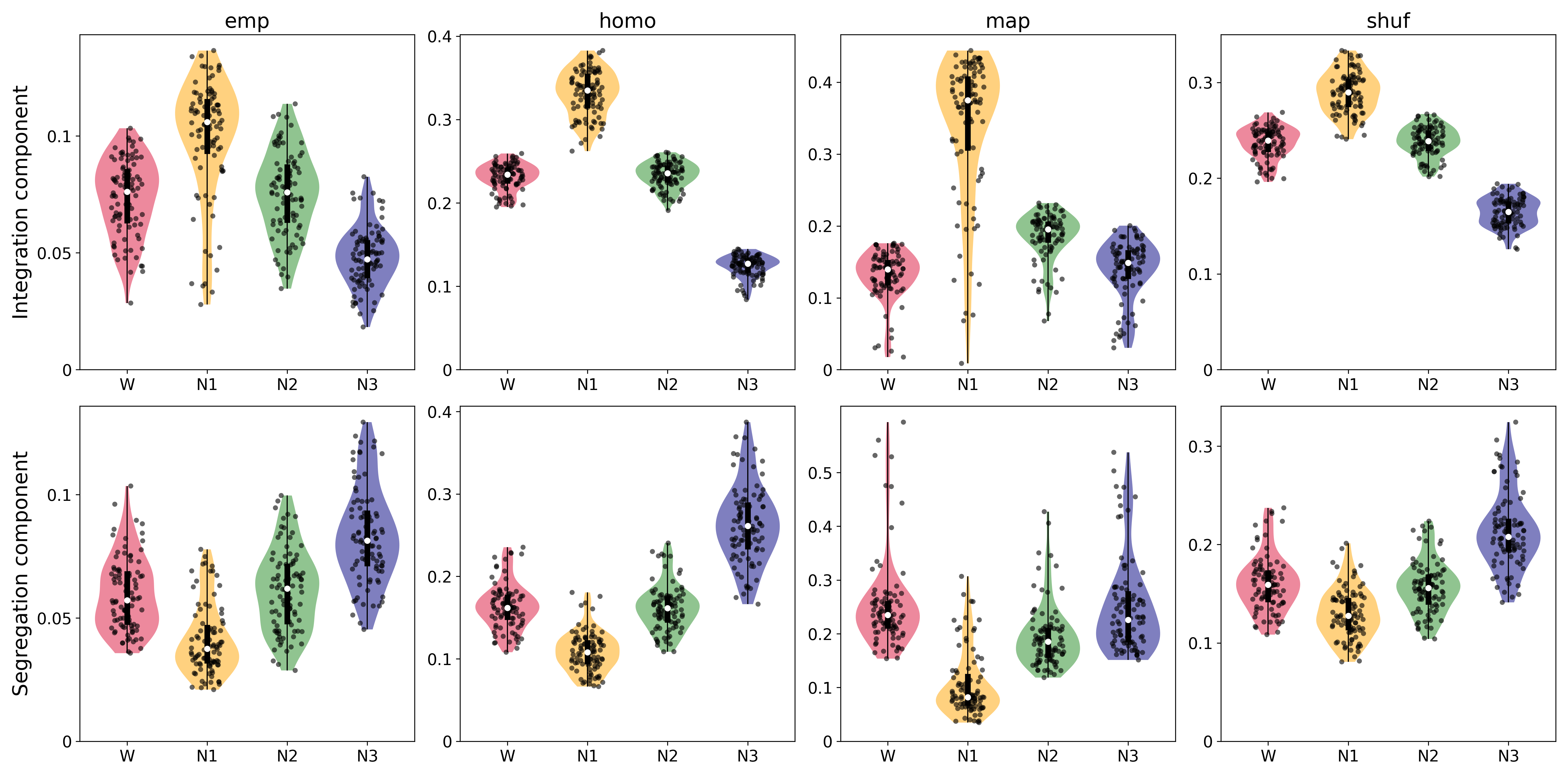

Supplement: S2 Fig — Each point is a brain area, averaged across individuals (empirical) or seeds (simulated, N=50 seeds). (TIFF) [file pcbi.1012852.s002.tiff]
