# Supplementary material for: Noradrenaline and acetylcholine shape functional connectivity organization of NREM substages: An empirical and simulation study
Source: PLoS Comput Biol. 2025 Oct 28;21(10):e1012852. doi: 10.1371/journal.pcbi.1012852 (PMC12585100; doi:10.1371/journal.pcbi.1012852)
Supplement: Attachment — Submitted filename: Response to reviewers 1.pdf [file pcbi.1012852.s003.pdf]

Reply to Reviewers
Noradrenaline and Acetylcholine shape Functional Connectivity organization of NREM substages: an empirical and simulation study

Fernando Lehue, Carlos Coronel-Oliveros, Vicente Medel, Thomas Liebe, Jörn Kaufmann, Sebastián Orellana, Diego Becerra, Enzo Tagliazucchi, Patricio Orio

June, 2025

We appreciate the positive response of the reviewers and thank for the comments and suggestions that indeed improved our manuscript. In the following, we provide point-by-point responses to the concerns raised.

Reviewers' comments:

Reviewer #1: This study explores how the neuromodulators Acetylcholine (ACh) and Noradrenaline (NA) influence brain connectivity during NREM sleep stages using a whole-brain model. The authors present a novel approach by integrating ACh and NA into a model informed by an empirical brain connectome and neurochemical maps. They identify key changes in functional connectivity (FC), such as decreased LC connectivity with the cortex in the N2 and N3 stages, and reduced BF connectivity with the cortex in N3. The study also reports a transition to a more integrated FC in N1 and a more segregated state in N3 compared to wakefulness. The model adjustments underscore the importance of region-specific neurotransmitter effects on brain connectivity.

The manuscript is generally well-written. I have made several comments listed below.

We thank the reviewer for this positive feedback.

Major: Is it possible to model other key neurotransmitters in relation to BOLD fluctuations? For example, GABA, which increases after NREM sleep compared to wakefulness and changes in the opposite direction of ACh and NA.

We agree with the reviewer in that the role of other neurotransmitters would be worth testing with a similar strategy. In particular, the increase in GABA which has been also extensively described. Although we are aware of another work that modeled variations in GABA as changes in the input-output slope of the inhibitory population (Luppi et al., 2022), we consider that the most direct way to implement it would be to vary the recurrent a_{IE} (inhibitory-to-excitatory) weight of the nodes. However, this is not directly feasible using our model, as the term a^{IE} is dynamically adjusted in order to reach an average level of activation of the excitatory populations (i.e. E_i approaches ρ^E, Eq. (3)). In other words, the level of GABA is an output of our model, not an input.

In any case, this comment led us to examine the level of inhibition of the areas of our model, by looking at the final values of a_{IE} (Figure 1 of this response letter). These were obtained by running the model in the different optimals that we found in our main sweep (obtained in the sweeps of SM1 and also displayed in Fig. 3 of the main manuscript), and comparing the distribution of a_{IE} across modalities and states. We found that when including the map, the a_{IE} values were higher in NREM modalities than in W (Figure 1A, top). Also, W showed lower a_{IE} values with the map than without it (homo and shuffled), while the opposite was true for NREM stages (see them grouped by stage in Figure 1, bottom). Note that these distributions closely resemble the variation of G in our model, and this is expected since a higher coupling makes the nodes require a stronger local inhibition to maintain

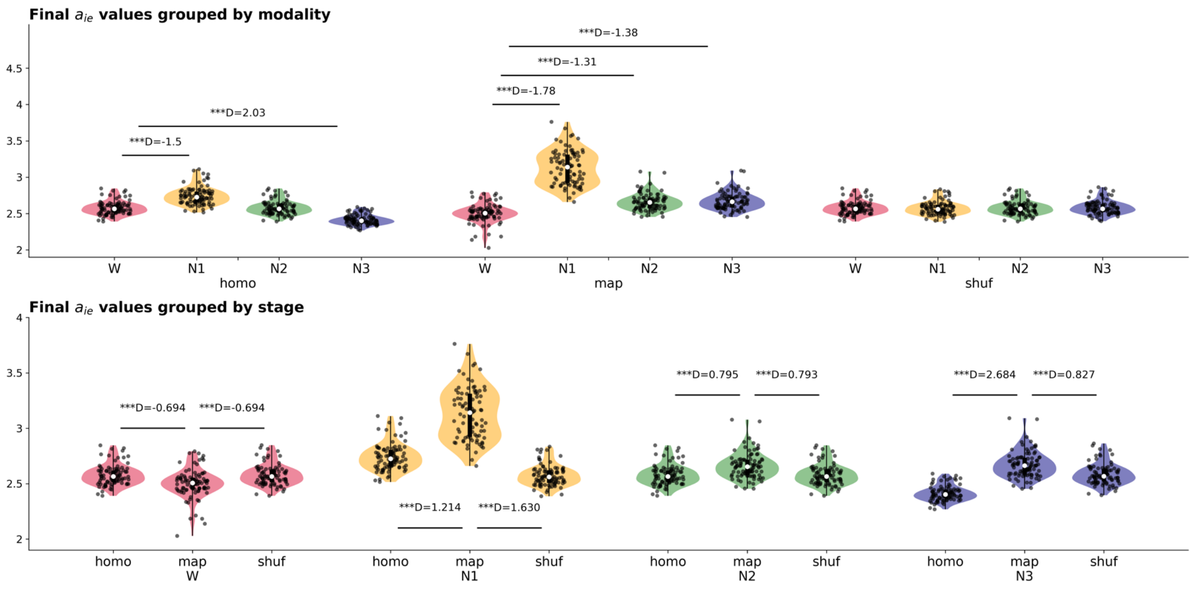

Figure 1: Final a_{IE} values attained by the model, grouped by modality and stage.

the excitatory variable E_i close to the target ρ_E. To control by variations in coupling, it would be necessary to understand the relationship between a_{IE} and node strength, which is complex and beyond the scope of this work. In the future, a suitable model with an adjustable inhibition parameter could be used, where a GABA modulation map can be included.

To ensure that the masks for BF and LC are appropriately chosen and positioned, they should be displayed on the BOLD data of a representative participant.

We understand the concern of the reviewer and we have added a panel to illustrate the positioning of the BF and LC in MNI space in panel C of Fig1 in the main manuscript, and also projected their positions on the BOLD data of a representative participant as a supplementary figure.

Lines 194-196: How did the authors decide on the parameters for δG and $\delta\gamma$? In addition, δG for N2 in the text is different from that shown in Fig. 4. In the heterogeneous modulation analyses, it would be useful to show the δG and $\delta\gamma$ maps across the brain.

We apologize for choosing words that may have been confusing. The values of δG and $\delta\sigma$ were chosen as the parameters that minimized the euccor metric in the sweeps as stated in the manuscript, and available as supplementary figures. Regarding the δG and $\delta\sigma$ across the brain, they basically correspond to the modulation maps re-scaled according to the 'global' δG and $\delta\sigma$ values. The maps are now included in Figure 2, that outlines the modeling framework.

Fig. 5C: Is it possible to compare the similarity among states for the same modality, e.g., the map strategy? It seems that the correlations between simulated and empirical integration (segregation) in N2 are the lowest. I would appreciate it if the authors could comment on this. Similar concerns raises for Fig. 3C.

This is a sharp observation that invites a closer look at how to interpret the fitting procedure. We measured the similarity of all simulated integration/segregation profiles to the rest of simulated and empirical ones (see Figure 2 in this letter), and we found that simulations with or without map were more similar between them than to their corresponding empirical counterparts. This is to be expected, because all simulations are obtained from the same model. The point we wanted to make was how the inclusion of the map makes the model have an integration/segregation profile that is closer to the empirical one than a naive model with no map or a shuffled one. This result could have been different if we directly fitted our model to the empirical integration/segregation profile, but considering that this was recovered from the fitting to the static FC of the simulated signals, we wanted

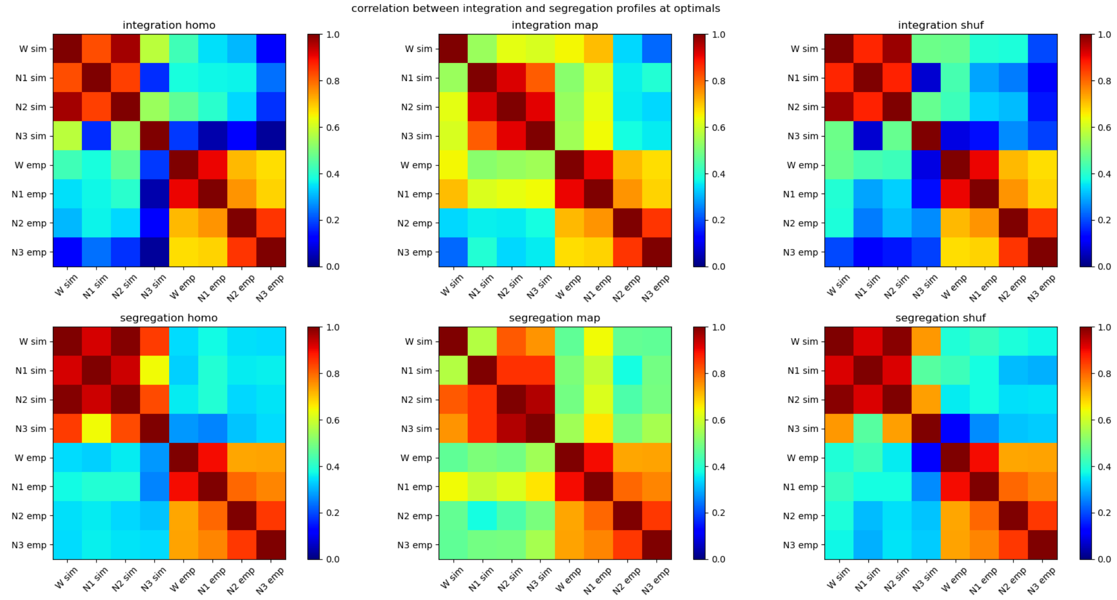

Figure 2: Correlation between the Integration (top row) and Segregation profile (bottom row) of all simulated and empirical FC matrices that we analyzed. Pearson correlation was calculated over the 90-valued vectors of integration/segregation per area, obtained after averaging over seeds (simulated) or individuals (empirical).

to show how other empirical observables can be improved even when not explicitly optimizing for them.

Regarding N2, it is the stage that our model fitted worse. N2 is characterized by the presence of spindles, which have their origin in the thalamocortical circuit, whose more intricate characteristics might be necessary to model the differences in the thalamocortical connectivities that we observe in our empirical matrices, and that our model is not capturing here.

Minor

The full spelling of ACh and NA should be provided at their first appearance, i.e., line 22.

Thanks for noting this. It has been corrected.

Reviewer #2:

The paper of Lehue et al. investigates the role of ACh/NA modulation in shaping FC across different sleep stages. The authors leverage experimental fMRI data to tune whole brain mean-field model in order to explain FC changes by ACh/NA release and present model covering all major sleep stages in humans. They show interesting point that using heterogenous map of neuromodulatory release based on presumed LC/BF connectivity improves the fit to experimental data, thus suggesting the role of neuromodulation in shaping sleep FC dynamics. They also connect their story with a broader thread of studies showing ACh/NE influences on segregation/integration properties of the network.

I find that the paper is timely and on an important topic and although I have a major concern to what extent can be their manipulation called "ACh/NA modulation" I believe this paper is of interest.

These are my points with decreasing order of importance:

1) I have major concern to what extent are the changes of global coupling and slope of input-output function of excitatory population realistic proxy for the ACh/NA modulation, because the effects of both ACh/NA are very complex. E.g. authors defend NA modulation by referring to decades old slope of input-output function proposal, but lot have been shown since then - there is

a maze of opposing (enhances/reduces syn. transmission) effects based on alpha vs beta receptors, region identity (PFC vs rest), layer sensitivity, etc which won't be covered by simply estimating map/connectivity from LC; the case of ACh won't be less complex. I am happy to be proved wrong, but covering all this looks beyond the scope of this work, so it seems that the casual reader should be warned in the beginning of the paper what "ACh/NA" effects actually translate to and that certain critical stance is warranted, instead of unsupported claims that ignoring specific receptors effects does not change general effect of tonic levels of ACh/NA (l.270).

We thank the reviewer for this important and well justified concern. We fully agree that the neuromodulatory effects of both noradrenaline (NA) and acetylcholine (ACh) are highly complex and depend on many factors—including receptor subtype, brain region, cell type, and cortical layer. The goal of our study is not to provide a mechanistic model of ACh or NA action, but rather to investigate whether their large-scale spatial distributions can account for systematic changes in FC across sleep stages. We have revised the text (particularly around line 270 and in the Introduction) to make these simplifications clear to the reader.

For NA, our assumptions build on the GANE (glutamate amplifies noradrenergic effects) framework, which, despite its simplicity, continues to be used in recent studies to interpret both human and rodent data (see for example Elliot et al., 2025 in humans and Haubrich et al., 2025 in rodents). We recognize that modulating the slope of the input-output function as a proxy for noradrenergic gain control does not account for the full diversity of tha actions of NA. We also agree that our assumptions for ACh are even more simplified, as in this work we implemented cholinergic modulation as a reduction in global coupling, inspired by its proposed role in enhancing local processing and reducing long-range correlations. We acknowledge that this is a highly reductive representation and do not intend it to capture the full spectrum of ACh's effects.

We now clarify these limitations more explicitly in the manuscript and have added a note in the Introduction, Brain Maps and Discussion sections.

2) There is a certain tension in rodent literature between traditional view that NA is reduced during NREM and more recent experimental work correcting this at several fronts - a) NA is indeed somewhat weaker in neocortical brain areas, but still overlaps with values at wake, b) for some regions (sensory thalamus) NA levels are even higher in NREM than in quiet wakefulness c) NA level is not tonic, but dynamic

To what degree presented results hold, if a) NA reduction in NREM is very slight b) oscillates in infraslow regime?

We thank the reviewer for these insightful observations.

Regarding the first question, we agree that the reduction of NA during NREM may be modest and region-dependent, and therefore we think that our main result is about the spatial distribution of NA modulation, as adding it based on LC projection patterns (through the GANE model related assumptions) improves the fit between simulated and empirical FC matrix beyond the effect of a randomized map. This suggests that the spatial heterogeneity of NA modulation may be just as functionally relevant as its overall tonic level. We see this as an important result in itself, supporting the idea that neuromodulatory gradients play a key role in shaping large-scale network connectivity during sleep.

For point b), we acknowledge that NA levels may indeed be higher in thalamus during NREM compared to quiet wakefulness. Our modeling framework does not capture this regional exception, and instead reflects long-timescale, mainly cortex-defined state-dependent averages (as the majority of the nodes are cortical). Therefore, our results should be interpreted as coarse-grained, cortical levels of NA, rather than as evidence for uniformly lower levels in all regions during NREM. In the future, thalamic specificity and infraslow fluctuations of neurotransmitters could be included in whole-brain models of brain activity. It must also be noted that, due to the limited temporal resolution and discontinuous nature of our fMRI data, our approach cannot resolve infraslow fluctuations in NA tone.

Finally, we would like to note that translating findings from non-human animals to humans must be done with caution. For example, the relationship between SWS delta power and spindle density differs across species, being positive in cats and mice, but negative in humans (as in Krishnan et al., 2016 (https://doi.org/10.7554/eLife.18607)).

These interspecies differences highlight the need for human-specific models and interpretations, which is what our work aims to provide.

Words of caution were added in the discussion about the interpretation of cortical and subcortical nodes.

I see the comment in manuscript (l. 442) that LC- thalamic connectivity needed artificial decrease of strength. Is there some possible connection with point b)?

Possibly, if the model had a proper model of the thalamus, the LC map could have been fed as it was without changes. However, all the nodes in our model are "cortical", and we had to make this adjustment to avoid the possibility that a node activity saturates. If this was not done, even a small variation of the sigma parameter would have had an effect that was practically exclusive to the thalamus, which would be difficult to interpret when our thalamus is modeled the same as the rest of the brain areas. We also comment this limitation of our work within the Discussion.

3) Data availability issues:

a) Authors claim fMRI dataset is available in Tagliazucchi et al., 2012. I could find the paper describing results, but no data. Is the availability statement misleading or can the authors provide better pointers where the experimental data are deposited?

We thank the reviewer for noting this. We uploaded the BOLD time series of the individuals that were used in this work in this work to Zenodo, available here. This is now also mentioned in the manuscript.

b) It looks to me that in the current form the simulation code at github is not reproducible and important private modules/files are missing (e.g. BOLDModel, "integration_segregation/", "RSN_AAL_Enzo.txt", etc). If they are not private, access to them should be described in dependencies of the code.

c) The connectome itself looks present after some digging, but should be better exposed. It would be nice if the files with a) global connectome b) maps/connectivity from LC/BF to the cortical sites are explicitly described/offered - either in methods or in github readme. Especially not so common explicit LC/BF -¿ cortex connectivity/maps in AAL could be useful for the modelling community.

The github repository has been reestructured, everything necessary to run the codes is available now.

Minor points:

- Do authors have a sense how much is using both hemispheres fundamental for the present results (i.e. if instead of artificially amending homotopic connections between hemispheres, only single hemisphere was used)?

Modeling both hemispheres allows us to account for the large scale interhemispheric interactions, which are particularly prominent in resting state fMRI. We aimed to preserve this and enhanced the goodness of fit of our FC matrices by symmetrizing homotopic connections in our structural connectivity (SC) matrix.

That said, we agree that assessing the extent to which our results depend on the bilateral model is an important direction for future works. This would require a couple extra considerations; given that almost all nodes would receive a lower input from the rest of the network, implementing a stable and physiologically realistic single-hemisphere model would require careful reparameterization of the SC and global coupling, which is nontrivial. Moreover, a single-hemisphere model would raise the additional challenge of how to incorporate the LC, which consists of a bilateral pair of nuclei with widespread, asymmetric projections. Our current assumptions do not make this decision and rely on a bilateral representation of NA and ACh modulation (through the parameter assumptions), and adapting a whole brain model to a unilateral model would require additional anatomical constraints and most likely alter the interpretation of all results.

While we have not run such simulations within the scope of this study, we believe that the qualitative nature of the role of neuromodulatory gradients in shaping FC would remain.

- I could not find explicit description how were the structural connections from LC to the rest of AAL areas established in methods (though I found relevant reference later elsewhere in the manuscript). Glancing over Liebe et al. paper suggests they do not seem to provide it in the right template(?).

Thank you for noting this. Now we include the appropiate clarification in the manuscript, in the Brain Maps section of Methods.

- The whole discussion section "Sleep as a model of consciousness" reads somewhat superfluous and I had difficulty to connect it with the actual results of the paper.

We agree that some sections and claims were off topic and restructured that part from a section to a short paragraph that comments on the implications for consciousness and dream research.

- The numbering of both figures/their panels does not fit the order they are introduced in the text and forces the reader to do chaotic jumps (e.g. 1B,1A,1C,5B,5A,2,3B,3,4,3C,5C,...)

Thank you for noting this. We have rearranged the mentioning of the figures, and reestructured the order of the sections (Integration-segregation now comes after simulation results) so now figures appear more sequentially (and naturally) when reading.

- Probably missing something obvious, but why are signs of +-ACh/-+NA opposite in Fig 3B, opposite y-axes?

This is a very important point. It is because an *increase* of ACh is associated to a *decrease* in G (long-range coupling). On the contrary, the increase of NA is related to an increase in the input-output slope. For transparency, we chose to display the variations in the parameter space instead of doing it in "interpretation" space. In our revised version of the manuscript, we have changed the label and renamed the axis as simply δ_G and δ_σ.

- Fig 3A - stage labels on top of the dots would be helpful.

- Caption of panel Fig3A contains description of violin plots, which should be probably elsewhere.

Typos:

l. 12 W -> (W)

l. 17 orchastrate

l. 159 - Figure -> Fig

l. 177 "the the"

l. 242 ")"

l. 347 N3, N1

l. 389 mm^3

l. 440 "("

l. 441 "document" sounds strange

We thank you for these observations, they have been corrected in the main manuscript.
