# Supplementary material for: Noradrenaline and acetylcholine shape functional connectivity organization of NREM substages: An empirical and simulation study
Source: PLoS Comput Biol. 2025 Oct 28;21(10):e1012852. doi: 10.1371/journal.pcbi.1012852 (PMC12585100; doi:10.1371/journal.pcbi.1012852)
Supplement: Attachment — Submitted filename: Response to reviewers 2.pdf [file pcbi.1012852.s004.pdf]

Reply to Reviewers
Noradrenaline and Acetylcholine shape Functional Connectivity organization of NREM substages: an empirical and simulation study

Fernando Lehue, Carlos Coronel-Oliveros, Vicente Medel, Thomas Liebe, Jörn Kaufmann, Sebastián Orellana, Diego Becerra, Enzo Tagliazucchi, Patricio Orio

September, 2025

We are happy to know that the reviewers deemed their concerns mostly addressed. There was only one minor issue that, as Reviewer correctly guessed, was not addressed by mistake.

Reviewers' comments:

Reviewer #2: I see the authors response to the major point #3 (Data availability) from the first review as inadequate, in particular:

1) Authors claim that git repository was restructured. I do not see any changes/commits in github repository so points 3b & 3c were not in fact addressed. Perhaps the authors forget to push the changes to the public repo?

2) New Zenodo deposit addressing 3a has restricted access (which is fine in the pre-accepted stage), but the available description of the dataset looks very insufficient for anyone to make sense of the deposited data. Given the repeated issues with data deposits for this paper I would appreciate Zenodo's hidden link for reviewers to review the deposit description details to decide whether they give the community chance to actually validate the paper claims.

We deeply apologize for not updating opportunely the repositories of our data and codes. The code repository found at https://github.com/vandal-uv/NREMmodFC was reestructured for clarity and reproducibility. Unfortunately, we just realized that the changes were committed to another fork, thus the given link contained the same old files. Now this is corrected.

Regarding the Zenodo deposit, we made a small change for clarity and consistency, related to the LC mask and one the concerns of Reviewer #1. Because of Zenodo policies, we had to create a new repository that can be found at https://zenodo.org/records/16755776. Unfortunately, the outdated github entry still made reference to the old Zenodo entry (now made private).

Both github and Zenodo repositories are now correctly indicated within the Additional Data in the submission system.
